# Genome Sequence of a Canadian Vibrio parahaemolyticus Isolate with Unique Mobilizing Capacity

**DOI:** 10.1128/genomeA.00520-18

**Published:** 2018-06-14

**Authors:** Audrey Bioteau, Kévin Huguet, Vincent Burrus, Swapan Banerjee

**Affiliations:** a Département de Biologie, Université de Sherbrooke, Sherbrooke, Quebec, Canada; b Bureau of Microbial Hazards, Health Canada, Ottawa, Ontario, Canada

## Abstract

Vibrio parahaemolyticus is a clinically significant marine bacterium implicated in gastroenteritis among consumers of raw or undercooked seafood. This report presents the whole-genome sequence of a unique strain of V. parahaemolyticus isolated from oysters harvested in Canada.

## GENOME ANNOUNCEMENT

Vibrio parahaemolyticus is autochthonous in estuarine environments around the world and is detected in seafood from contaminated harvest sites. Since the 1950s, V. parahaemolyticus has been frequently implicated in cases of seafood-borne gastroenteritis and other related illnesses (1, 2). Most of the clinical isolates are known to express thermostable direct hemolysin (TDH) and/or TDH-related hemolysin (TRH), encoded by *tdh* and *trh*, respectively (1, 3, 4). However, clinical isolates lacking both *tdh* and *trh,* as confirmed by PCR, have been detected from several regions, including in recent years from Canada (5) and from the United States (6). The diversity and dynamics of V. parahaemolyticus strains testify to the adaptability of the species to a wide range of habitat and/or environmental challenges by virtue of its genome plasticity and resultant evolution (7). Changes can also occur via the processes of integration and/or conjugation of genetic material through horizontal transfer (8). These events can lead to the emergence of new pathogenic strains as well as evolution of the existing pathogens (7, 9).

Knowledge of the global distribution and epidemiology of V. parahaemolyticus, including its genomic profile, will help understand its impact on the human host and the marine environment. Here, we report the genomic sequence of an environmental V. parahaemolyticus isolate from oysters harvested in Canada in the summer of 2005 and sourced to Ladysmith Harbor, British Columbia, Canada.

V. parahaemolyticus strain S107-1 was isolated and characterized at the Health Canada laboratory by an in-house procedure, the details of which have been published elsewhere (10). An antimicrobial resistance profile (AMR) of the isolate was determined by Kirby-Bauer's disk diffusion method (11, 12). Whole-genome sequencing of V. parahaemolyticus S107-1 containing ICE*Vpa*Can1 was carried out from genomic DNA extracted from 2 ml of exponential-phase culture using the Gentra Puregene kit (Qiagen). PacBio RS II single-molecule real-time sequencing (PacBio SMRTcell) and *de novo* genome assembly were performed at the McGill University and Génome Québec Innovation Centre with the HGAP version 3 method. Annotation data for V. parahaemolyticus S107-1 were provided by the NCBI Prokaryotic Genome Annotation Pipeline (13).

Biochemical analysis using API20E diagnostic strips confirmed the isolate (S107-1) to be V. parahaemolyticus, which also tested positive by PCR for the presence of the species-specific marker, thermolabile hemolysin gene (*tlh*), while the known virulence markers (*tdh* and *trh*) for V. parahaemolyticus tested negative by PCR. This isolate was resistant to three antibiotics, ampicillin, streptomycin, and cephalothin, and showed intermediate resistance to piperacillin and kanamycin. Whole-genome sequencing analysis identified and assembled the two chromosomes of sizes 3,445,421 bp and 1,757,905 bp for chromosomes I and II, respectively.

The presence of an integrative and conjugative element (ICE), consequently named ICE*Vpa*Can1, was detected at the 5ʹ end of the *prfC* gene on chromosome I of the V. parahaemolyticus isolate S107-1. This element of the SXT/R391 family has a size of 81,255 bp and does not carry any predicted resistance genes.

### Accession number(s).

The complete genome sequences of the two chromosomes of V. parahaemolyticus strain S107-1 were deposited at GenBank under accession numbers CP028481 (chromosome I) and CP028482 (chromosome II).
